# Visual diplomacy in virtual summitry: Status signalling during the coronavirus crisis

**DOI:** 10.1017/S0260210521000607

**Published:** 2021-10-19

**Authors:** August Danielson, Elsa Hedling

**Affiliations:** 1 Department of Government, Uppsala University, Sweden; 2 Department of Political Science, Lund University, Sweden

**Keywords:** Summit Diplomacy, Status, Signalling, Visual Politics, COVID-19

## Abstract

On 26 March 2020, the leaders of the Group of twenty major economies (G20) convened in an emergency virtual meeting to discuss the extraordinary situation facing the world. Virtual summitry provided a stark visual contrast to the traditional staging of modern multilateral diplomacy – leaders were suddenly responsible for their own staging, leaving them with new opportunities to create a favourable impression of how they, and their respective state, would be seen. Taking the disruption of virtual summitry as a starting point, we focus on the resulting new opportunities for visual diplomacy. We draw on the symbolic interactionism of Erving Goffman and we argue that status signalling in this context was based on a shared understanding of the symbols and resources that have social value in the interaction order of summit diplomacy. Based on a visual analysis of 51 photographs from the G20 video conference, we find that the visual performances during the extraordinary meeting reflected evident, but not necessarily intentional, attempts at status seeking. The article thus contributes to an increased understanding of how visual performances contribute to uphold status distinctions in multilateral diplomacy.

## Introduction

Striving to be seen in a favourable light is one of the reasons why state representatives participate in the highly institutionalised and ritualised practices of multilateral diplomacy.^1^ To be ‘seen’ in this context has partly to do with projecting a certain image of the state, but is equally about seeking recognition from other states that one is a legitimate participant in one of the essential social practices of high diplomacy. When the cancellation of physical summit meetings following the coronavirus crisis caused ambiguity over decorum and etiquette, the resort to virtual summitry provided new ways to be seen, and thus also new opportunities to create favourable impressions. For this reason, virtual summits quickly became one of the main ways for states to engage in ‘visual diplomacy’ – the ways in which diplomacy depends on visuality,^2^ visual artefacts,^3^ or visual staging.^4^

In the particular context of summit diplomacy, visual diplomacy concerns the ways in which states seek to influence how they are seen at summits. While modern-day summits are characterised by an elaborate protocol of etiquette – the advantage of which is to create common ground across different cultures and diverse political aspirations^5^ – this protocol is also part of a shared language that can be used to send messages intelligible only by those who have already internalised the diplomatic code.^6^ Summit meetings are therefore important arenas for state representatives to create favourable impressions of themselves that are recognised by others according to this diplomatic code. Performances such as ceremonial arrivals and high-profile handshakes can therefore be seen as both symbolic interactions and staged ‘visual spectacles’.^7^ As such, these routines contribute to uphold the shared rules and conventions of summit diplomacy – in sociological terms, ‘the interaction order’ – within the given social community.^8^ Given the routinised character of summit diplomacy, what states and their leaders do in this context is highly dependent on these routines being upheld.^9^ In this article, we study how states act in the event of a disrupted interaction order, specifically the visual diplomacy that resulted from the swift shift to virtual summitry in the wake of the COVID-19 pandemic.

On 26 March 2020, the leaders of the Group of twenty major economies (G20) convened in an emergency meeting to discuss the extraordinary situation facing the world. By then, the rapid spread of the virus had led to the shutdown of entire states and travel restrictions and quarantine protocols meant that the extraordinary G20 summit needed to be held virtually. In the months that followed, virtual summits gradually became the ‘new normal’ in multilateral diplomacy.^10^ This disruption of routinised summit diplomacy placed world leaders in unusual visual frames. Instead of photographs of leaders lining up on a stage for the traditional summit ‘family photo’, summits were communicated to the world through the multiple gallery views of video conference tools. Virtual summitry thus provided a stark visual contrast to the traditional staging of modern multilateral diplomacy – leaders were suddenly responsible for their own staging, leaving them with new opportunities to create a favourable impression of how they, and their respective state, would be seen in a ‘synthetic situation’.^11^

Taking the disruption of virtual summitry as a starting point, we focus on the resulting new opportunities for visual diplomacy. We argue that the absence of previously routinised visual performances placed the participants of the G20 meeting in a position to influence how they were seen and thereby reproduce, or potentially challenge, the rules and conventions of summit diplomacy by appropriating their virtual participation. The destabilisation to otherwise scripted routines of multilateral diplomacy provides us with a rare glance of the frames of social participation in the summit diplomacy context. In particular, by giving attention to the ways in which the participating states signalled status during the virtual summit, we can learn more about how visual performances contribute to uphold status distinctions in multilateral diplomacy.

In the following, we build on previous scholarship on visual diplomacy and engage with the symbolic interactionism of Erving Goffman^12^ to consider the ways in which visual performances during summit diplomacy contribute to uphold an interaction order of status seeking. Drawing on constructivist approaches to International Relations (IR), we expect such staged diplomatic performances to be concerned with the projection and recognition of status.^13^ Visual performances enable status to be signalled in efforts to influence how states are seen in relation to the other participants as efforts of impression management.^14^ As a result, by analysing these visual performances, the article contributes to a more fine-grained understanding of the resources, symbols, and signals that are perceived to have social value in multilateral diplomacy in general, and in the context of summits in particular.

In contrast to previous research on visual diplomacy and diplomatic signalling, our approach seeks to describe how visual performances reflect common understandings of status symbols, markers, and resources, and in this sense reflect patterns of social interaction and conformity in international politics. While diplomacy is an inherently visual practice,^15^ empirical demonstrations of how this visuality matters have thus far been limited and often restricted to analyses of public diplomacy.^16^ Further, influencing one's perception in the interaction with others through visual cues is one way in which states can convey signals in international politics. Previous attempts to further theoretical approaches to signalling have, however, been preoccupied with the intentions, mechanisms, and transactional effects of signalling.^17^ We therefore contribute to the burgeoning literature on visuality in IR and diplomatic signalling by offering a framework in which visual performances of diplomacy can be studied in relation to the patterns of social interaction they contribute to uphold rather than the perceived credibility of the signal itself.

The article first situates visual diplomacy as historically relevant in the practice of multilateral diplomacy and diplomatic signalling, and traces its role and development in the digitalisation of diplomacy. We suggest that summit meetings present highly valued opportunities for status signalling, and therefore contribute to uphold the interaction order in this context. We argue that these opportunities have been amplified in the digital age. In the second part, we develop our interactionist approach to visual diplomacy in virtual summitry, which we operationalise as efforts of creating a favourable impression in a new situation through status signalling. In the third part, we trace status signalling in the ways in which the summit context was staged by the participating states through their choice of backdrop, actors, venue, and props in the photographs from the G20 virtual summit. These four dramaturgical elements allow us to identify the ways in which states choose to signal status through their visual performances when given the rare opportunity to do so. Our study draws on a dataset of 51 photographs depicting the state leaders’ participation in the summit. In the final part, we discuss our theoretical approach in relation to the disruption caused by the forced shift to virtual summitry and assess the significance of our findings for future research.

## Visual diplomacy, signalling, and digitalisation

Visual performances are important to the recognition of significant moments in international diplomacy.^18^ We know this primarily from historical photographs and the vivid descriptions of seminal events of war and peace. But visual performances are more than reflections of politics, they are inherently political because they frame what is thinkable and doable.^19^ Some international events are more visual than others. When international leaders sign a peace treaty, the occasion is ceremonial, a staged procedure that will be captured by photographers and shape the imagination of history.^20^ It is through these visual representations and symbols that expectations of appropriate behaviour during such monumental events are shaped.^21^ These performances are expected to the extent that they are often unreflected reproductions and therefore depoliticised, representations of previous political events.^22^ In this regard, disruptions such as interfering events or failure to act appropriately, can influence future expectations. While such moments are perhaps rare, regular photo opportunities, television appearances, state visits, and summit meetings also present opportunities for leaders and diplomats to perform ‘visual diplomacy’ by being seen in performances that we recognise as diplomacy.^23^

Costas M. Constantinou^24^ has defined visual diplomacy as the ‘ways and means by which images – still or moving images, often in combination with verbal comment or aural background – are used by plural diplomatic actors to transmit ideas to audiences, producing and circulating meanings that serve particular purposes, with the aim of influencing, shaping and transforming relations between actors and across publics’. This is a broad definition that encompasses many areas of visual diplomacy. Visual performances, however, in contrast to general visual representations of diplomatic events, allude to the specific role that visuality plays in the recognition of diplomatic practice. While visual performances are always accompanied by or complemented with discourse, they are also contrasted with spoken and articulated modes of communication, and therefore politically significant in their own right.^25^ Visual performances may, for instance, suggest alternative interpretations of a diplomatic event by displaying tension in the body language between leaders, despite the absence of such discourse. Visual performances may also complement discourse by serving other political purposes. For instance, summits meetings during the Second World War enabled leaders to reassure their publics by being seen together in recognised behaviour of diplomacy.^26^ An important part of these meetings were therefore the ceremonial photographs taken of the ‘Big Three’ – Roosevelt, Churchill, and Stalin.^27^ While these photographs are representations of diplomatic events during which what was said and written certainly carried meaning, the photographs themselves and the performances that they captured have also had a distinct impact on international politics.

Modern-day international summitry includes a far greater number of leaders that meet frequently, which has led to summits becoming increasingly formalised – including rituals such as staged photographs of participating leaders. Frequent diplomatic summits and regular media coverage have made these events even more visually coherent: there is careful attention to etiquette and ceremonial appearances in front of the cameras, and such occasions are therefore also important demonstrations of hierarchy and rites of passage for newly elected leaders. This increasingly ceremonial and symbolic emphasis on international summits has to some extent surpassed their political purpose. Jan Melissen^28^ has suggested that it is the ceremonial aspect rather than the substance of the negotiation that makes a multilateral summit ‘real’ to the general public. They provide opportunities for visual performances in the transmission of ideas between actors (interstate dialogue) and across publics (public diplomacy, or diplomatic communication aimed at domestic or international audiences).^29^ In this capacity, visual diplomacy is, therefore, a means of diplomatic signalling, a practice of packaging diplomatic messages through extra-linguistic forms of communication that complement, illuminate, or supplement language.^30^ During international summits, leaders interact with multiple other leaders using signals while also balancing both domestic and international public opinion through the news media's reporting.^31^

Although diplomatic signals often target multiple audiences and are therefore performed with a clear motive (or motives) in mind, not all signals are deliberate. The fact that many world leaders lack diplomatic training adds to the ambiguity of signals during summit meetings. Leaders may be signalling something they are not even aware of. In addition, unexpected events and uncertainty may lead to unintended signalling when actors make different interpretations of appropriate behaviour. Hence, diplomatic signalling occurs whenever one actor displays behaviour that is seen, perceived and interpreted by another, regardless of whether it is spoken or intended or even part of the actor's conscious awareness.^32^ This means that signalling also encompasses non-behaviour – a signal may for instance come in the form of not showing up to a summit meeting or being seen to perform a ‘handshake snub’. Practices of signalling hence depend on expectations of diplomatic behaviour in a given context. In routinised practices of diplomacy, signals are therefore best analysed in relation to the social conventions that determines expectations of recognised diplomatic behaviour. Yet many of these practices of signalling, through performances based on a specific interaction order, slip under the radar in scholarship on costly signals.^33^ We therefore suggest that more attention to visual performances of diplomacy may offer opportunities to deepen our understanding of signalling as social practices that may both reproduce and challenge international politics in their specific context.

Attention to visual diplomacy, in the understanding we have suggested, is further relevant to IR in light of increasing digitalisation of diplomacy,^34^ not least because of the fact that social media has made diplomacy more visually saturated.^35^ In the context of summit diplomacy, press photographs of leaders lined up in front of summit banners have been supplemented by self-reporting in popular strategies of online public diplomacy. In this context, visual performances contribute to a pluralisation of diplomatic practices.^36^ When leaders post ‘selfies’ of themselves with other leaders during high-profile events, this is just one example of new opportunities to proactively manage states’ reputations.^37^ Video clips from summits, for instance, often ‘go viral’. One famous example of such a video clip depicts the US President, Donald J. Trump, pushing his way to the front of other leaders during a 2017 NATO summit. While the intent behind such forceful body language is unknowable, Trump may have been attempting to challenge the social conventions by signalling assertive leadership to both his peers and the general public. This occasion illustrates the potential influence of being seen in unpredicted behaviour, caused by Trump's untraditional style of diplomacy, that we have access to because of the increased occurrences of visual performances during international summits. Trump's body language tells us something of both the traditional interaction order he is challenging and the new one he may contribute to create. This example also illustrates what Constantinou has suggested is a lost sense of control of the diplomatic plot through the visual opportunities of social media in a ‘post-protocol era’.^38^ We thus suggest that there is ample opportunity to develop analyses of visual diplomacy and that doing so may advance our understanding of social practices such as status seeking in seemingly ‘new’ diplomatic situations.

## Status in multilateral diplomacy

In the context of international diplomacy, visual performances of a nation and its leader help shape the intersubjective reality of a state's constitutive properties – in other words, whether we perceive a state to be ‘strong’, ‘weak’, ‘rich’, ‘poor’, and so on. Above all, visual representations of a state, and particularly how it is represented in relation to other states, help construct its diplomatic status in the international system.^39^ In this sense, how a state is visually represented and how such representation is performed, matters not just because it can have a causal impact on diplomatic outcomes, but particularly because it helps determine what a state *can* do in relation to its positioning among other states.^40^

In contemporary IR research, status has been defined as ‘collective beliefs about a given state's ranking on valued attributes’, such as ‘wealth, coercive capabilities, culture, demographic position, socio-political organization, and diplomatic clout’.^41^ In this sense, status is a favoured source of capital in international diplomacy and therefore central to the understanding of the rules of conduct in diplomatic interactions. Status is, however, at the same time ‘collective, subjective and relative’ – it is intersubjectively created and defined, as well as intrinsically hierarchical.^42^ States may strive to increase their social position on the international hierarchical scale for different reasons but, as Anne L. Clunan^43^ argues, it is ‘in a nutshell, about who is endowed with legitimate social power: authority’. In other words, increasing a state's status typically provides it with more power to create, adapt, and interpret the existing rules of the game of the international system. In this sense, *status seeking* is the pursuit of the legitimisation of domination, or a condition where actors that have less authority either approve of or at least accept the claims of the actor(s) with authority.^44^

From this perspective, it is by being recognised as a legitimate status holder by others that a state's status is defined – not by having certain state attributes. Having a high degree of status in world politics is therefore often defined by path dependence.^45^ The distinction between a state with high status and one with low status eventually becomes conventional, and even internalised by those that are least benefited by the ruling social hierarchy.^46^ However, this does not mean that states simply accept their fate and their position in the contemporary status order; on the contrary, the literature on ontological security has highlighted the role that insecurity regarding one's self-identity (as well as the feeling of shame that follows this insecurity) often drives states to pursue a higher status position in the international society.^47^ In addition to highlighting ontological insecurity as a driver of status-seeking, this constructivist/practice perspective also underlines the inseparability of a state's status and others’ perceptions of that status, which assumes that states are predisposed to act in line with the identity that is constituted by their status in the international system.^48^ This ontology can be contrasted with the notion that a state's self-image can be distinguished from the collective's view of that state's status and identity, which is more apparent in studies that apply social identity theory.^49^ Leaving aside the question of whether states have agency to affect their status position, collective and subjective perceptions of relative status seemingly matter a great deal to the patterns of interaction between states in international diplomacy.

## Status seeking and interaction order

In order to consider how the visual performances during the virtual summitry that arose from the coronavirus pandemic reflect status seeking, we turn to the symbolic interactionism of Erving Goffman. First, in the summit context we assume that a state's perceived status is the most important factor in the creation of a favourable impression. For Goffman, all interactions are opportunities for actors to present themselves in ways that will have social value in the given situation.^50^ Interaction thus creates the order according to which others will interpret your behaviour and hence your status.^51^ Since we have suggested that the struggle for status in IR is a relational process, interactions between states can be regarded as processes of status seeking behaviour.^52^ It is in this context that multilateral diplomacy, conducted through routines, in different ways contribute to uphold the hierarchical system where states struggle for recognition (by others) of their status. Further, an interaction order amounts to a shared understanding of appropriate behaviour – in Goffman's terms, ‘performances’ in relation to others. A performance is ‘the activity of a given occasion which serves to influence in any way any of the other participants’.^53^ Routinised diplomacy such as summit meetings are institutionalised interactions between states with a shared understanding of how actors should perform in ways that would reflect status. By participating in international summits, states and their diplomats act according to a shared definition of the situation – an interaction order that guides this struggle through scripted performances.^54^ Such scripted performances can be uncovered through attention to the would-be ‘idealised image’ of each participant. In the context of multilateral summits, state leaders might act competently or incompletely, depending on whether they live up to the rules and norms of appropriateness that exist within the ‘club’ of state leaders. This interaction order is also partly autonomous from the distribution of resource in the international system.^55^ In other words, even a leader from a state with significant material resources can perform incompetently in the eyes of other state leaders

According to Goffman, participants adapt their performances through ‘staging’ and ‘sign vehicles’ to present a version of themselves that will be superior to reality.^56^ Appropriate staging and sign vehicles are determined by the definition of the situation, the interaction order in which participants will seek to project their idealised image.

Since summit diplomacy is a highly institutionalised and ritualised practice of international politics, the interaction order for most given situations must be considered stable. This includes diplomatic visual performances, where summit diplomacy is an activity of interstate symbolic politics that contributes to visualising the state by allowing publics to ‘see’ its relationships with other states.^57^ State leaders and diplomats therefore know how to perform and how to present themselves in ways that will be correctly ‘seen’ by others. Still, even the most stable interaction order may be disrupted.^58^ Actors may seek to change the rules of conduct by producing new interpretations of a situation, for instance by being seen in unexpected behaviour (previously illustrated by Trump), but even then, wish to create a favourable impression of themselves within the framework of the pre-existing interaction order.^59^ Thus, even when an interaction order is destabilised, we can expect actors to engage in status seeking behaviour.

## Status signalling in summit diplomacy

Using Goffman's concepts of ‘interaction orders’ and ‘performances’ that are assisted by ‘staging’ and ‘sign vehicles’, we suggest that the interaction order of summit diplomacy is (visually) upheld through status signalling. In this context, some state attributes will be seen as having more social value than others, in the sense that they are linked to the role of a high-status actor in that setting.^60^ Again, this does not mean that having these attributes will necessarily lead to higher status, but simply that other states believe that they can increase their status if they present themselves as having them. A relevant analogy would be the *nouveau riche* who aim to present themselves as wealthy by purchasing expensive cars or clothes, without being recognised as such by other members of that social class.^61^ Those that perceive themselves to have low status in a group will thus be predisposed to increase their status by trying to achieve these attributes, or at least present themselves in an idealised image as if they already have them.

In order to identify status signalling in the virtual G20 meeting, the attributes that should be perceived as having social value within this social context must thus first be mapped. To do this, we draw mainly on the literature on summit diplomacy, signalling, and status in world politics. Since the safest option for a state entering a novel environment is to mimic other actors,^62^ it is reasonable to assume that states and state leaders will place roughly the same social value on the resources that generate status in a virtual summit meeting as those that generate status in the context of a physical summit meeting. In this sense, while the virtual setting allowed status seeking performances that would otherwise not be possible given the neutral venue norm (no opportunity for staging), we should still assume that the signalling that took place was based on the participants’ prior dispositions and shared understandings about the attributes that are perceived to lead to greater recognition if it would have been possible to signal them in a physical summit meeting. While there are arguably many attributes that will be perceived as increasing one's status in the context of summit diplomacy, we single out four attributes, resources, or factors that we believe should be perceived as having relatively high social and symbolic value when expressed through visual diplomatic performances in this context: reliability, trustworthiness, material capabilities, and continuity. As Vincent Pouliot points out, the social value of status resources are historically contingent and socially defined.^63^ Our choice of resources is thus a reflection of the relative degree of social value that we perceive them to have in the context of summit diplomacy today. While the status resources that have social value in terms of world politics in general, such as ‘military power, economic development, cultural achievements, diplomatic skills and technological innovation’^64^ are indicative of the attributes that should have social value in the context of summits, we should not assume that they are synonymous. For this reason, we specify why these four resources or attributes should be regarded as having social value in the context of summit meetings in conjunction with the description of each resource, as well as throughout the analysis.

In context of summit diplomacy, *reliability* should be understood as having a credible capacity to act competently over time. In other words, a leader that signals reliability also signals consistency and resolve, meaning the ‘belief that an actor is determined to achieve their goals and is willing to endure potential costs in pursuit of those goals’.^65^ A second resource of status in summit diplomacy is *trustworthiness*, or the quality of being perceived as consistently making honest and credible statements. Trustworthiness differs from reliability in the sense that the former is about being perceived as honest more generally while the latter is about having a credible capacity to act. In this sense, trustworthiness can be equated to sincerity.^66^ The third primary status resource in summit diplomacy is the perceived degree of *material* (economic or military) *capabilities* that a state leader has at his or her disposal. Even if we accept that the social value of status resources is socially defined,^67^ material capabilities still matter as long as we believe that they do – and states seemingly do believe that they matter.^68^ The final resource that can increase a leader's status is a sense of *continuity*, or historical capital. One of the three ways in which Max Weber argues that actors can lay claim to the right of legitimate domination is so-called ‘traditional authority’, which is based on ‘an established belief in the sanctity of immemorial traditions’.^69^ In the context of summit diplomacy, this could be done by promoting the historic economic or cultural superiority of a state, and in this sense marking the ‘normative difference’ between them and ‘uncivilised’ states.^70^ While this may often be done by referring to the ‘standard of civilisation’ that is associated with nineteenth-century Europe, it is not a practice that is limited to European states – even states outside of Europe may seek to contrast themselves to an idea of the uncivilised Other as a way to strengthen their self-perception as part of the West.^71^

In the following analysis, we use these status resources as a way to identify the ‘baseline’ of status seeking in the context of interaction order during summits. In other words, these are the resources that we would expect states and state leaders to draw on if they were able to seek status in an ordinary, physical summit meeting. In this sense, they can also be seen as constituting a part of the interaction order for staged performances of summit diplomacy. However, even though we make the case that these resources or attributes should be perceived by states and state leaders as having value, we do not go so far as to say that the actors who are signalling status are aware that they actually are sending signals. In our view, it is not necessary to assume that states do or do not have agency when signalling status. Either ontology may be accurate. In the context of this study, however, the important question is rather how signals are being sent, not whether state leaders are aware that they are sending them, why or to what effect. For this reason, we make no attempt to identify the intentions behind particular status signals, and instead present them as how we perceive that they would be reasonably understood by the other participating states and state leaders.^72^

## Status signalling during the virtual G20 meeting

**Figure 1.**
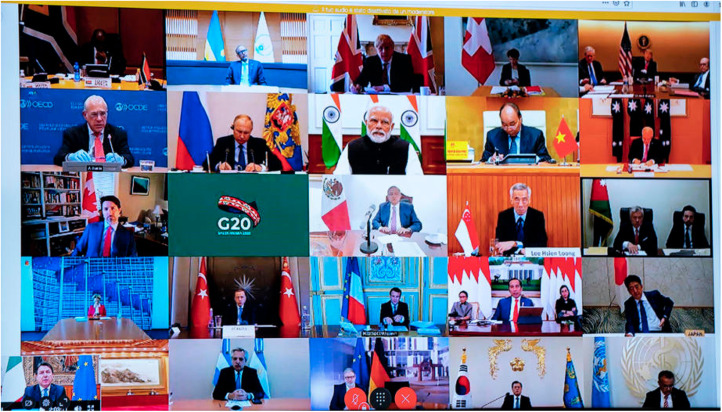
Photograph of a television screen at the Palazzo Chigi in Rome, showing the video conference between the G20 leaders. *Source*: Environmental Protection Agency.

As we have noted, the first virtual G20 meeting was a visual spectacle. In contrast to usual summit meeting rooms where the only visible symbols are national flags in the backdrop of the venue, the format of the meeting allowed the leaders of the world's twenty largest economies to sidestep the traditional summit rituals and use a wide array of non-verbal signals. Furnishings, paintings, and even the number and placement of advisors were all new elements of the social context of the summit, which made photographs of the meeting look more like a colourful quilt than a regular conference call (Figure 1).

This study was conducted by collecting 51 photographic depictions of the G20 meeting. A majority of these photographs were published by the official communications channels (websites and Twitter) of the G20 member states and the invitees who took part in the meeting. We collected photographs that pictured all twenty member states from as many angles as possible, including both the video conference view (the view that the other participants would have seen during the meeting) and the gallery view (the screen picturing the multiple participants). Our analysis was inspired by visual semiotics, which looks for signs in visual elements such as photographs. Semiotics is the study of signs and the way they acquire meaning, which is relevant to any study of diplomatic communication.^73^ The meaning of signs, however, depends on social conventions and we recognise the difficulties in differentiating between the visual signs in an image, which stresses the important role of intertextuality in the meaning making of signs.^74^ We were therefore inspired by the ‘critical visual methodology’ of Gillian Rose^75^ and approached the photographs from a discursive perspective in which we took the specific cultural significance, social practices, and power relations expected in staged procedures of international diplomacy into account. This method of analysis entails carefully examining both the content and intertextuality of images, the way in which the meanings of any discursive image depend not only on that single image, but also on the meanings carried by other images and texts.^76^ In practice, this meant that we mainly analysed the photographs by looking for different expressions of the status resources mentioned above, as we expect these resources to be central in the interaction order of summit diplomacy. For this purpose, our analysis is structured around four discursive elements of a diplomatic theatrical staging: *backdrop*, *actors*, *venue*, and *props*.^77^ These stage elements allow an analysis of how images are given meaning when constructing a ‘diplomatic stage’. In this sense, how the participating states chose to use these stage elements allowed us to trace the aforementioned status resources in visual performances of diplomacy. This section thus analyses the 51 photographs and discusses how status was signalled through the four stage elements.

### Backdrop

In the case of traditional summit diplomacy, the backdrop of a meeting is conventionally constituted by a row of the participating member states’ national flags to symbolise interaction between states.^78^ In this sense, flags contribute to visually uphold an interaction order between equal member states. The role of flag symbolism in international diplomacy, including summit meetings, can be seen as a continuing opportunity and responsibility to project the image of a nation.^79^ The virtual G20 meeting mimicked such use of national flag but stood out in terms of the variation in how states chose to project the nation's image in their respective backdrop (that is, the visual elements placed behind each state leader). While at least one flag was placed behind every participating leader in order to indicate which state they represented, there was a significant variation in how many flags were presented as well as how ‘noticeable’ they were (Figure 1). For instance, while states such as India and Indonesia chose to place as many as ten flags behind their leader, the majority of states – including the United States, China, and Japan, the three largest in terms of nominal gross domestic product (GDP) – only presented a single flag. Six states chose to place two national flags behind their leader, including the UK, Russia, and Turkey. The three participating EU member states, Germany, France, and Italy, each placed an additional EU flag behind their leader.

One interpretation of the decision to place a large number of flags behind a leader is that it is a signal of the relative importance of the nation and its national interests over the common or ‘international’ interest. Given the norm of ‘flag equality’^80^ in multilateral diplomatic settings – in other words, that the number and size of the participating states’ flags should always be equal as a way to symbolise the equality of states – the choice to place a large number of flags in the backdrop can be a way to signal the opposite message: some are more equal than others. Putting a great emphasis on a state's flag can also be a way to signal the status resource of continuity. Since a flag is one of the clearest symbols of the history of a nation, it can be used in staging by states to distance themselves from other states that do not have an equally long and prosperous history. While this was likely not the intended message when placing a flag behind a leader, it may still be perceived as such by the remaining participating states, especially those that are more insecure about their own identity as a member of the G20. Given that ontological insecurity should be most common among those that are on the periphery of group membership,^81^ it is thus reasonable to expect that the members with the lowest GDP would consciously or subconsciously choose stage the backdrop with a larger number of flags – and thus signal national pride – while the members with the highest GDP would have fewer and smaller flags.

While there was seemingly not a strong correlation between these factors, some states stood out. As mentioned, the three largest states only showcased a single flag, while states such as Indonesia (ranked 15^th^ in terms of nominal GDP), Australia (13^th^), and India (20^th^ in terms of GDP per capita) placed between four and ten flags behind their leader, indicating that ontological security/insecurity about one's historical capital and material capabilities may have had some effect on how states chose to stage their backdrop. However, the difference may also be explained by variation in national culture. For instance, Raymond Cohen^82^ has argued that matters of status and dignity are more salient in so-called ‘high-context national cultures’, such as China, India, Japan, and other Asian countries, as well as Saudi Arabia, Italy, and Russia. High-context cultures, a concept originally formulated by Hall,^83^ are distinguished by the importance placed on verbal and non-verbal cues, rather than the spoken word itself. In contrast, low-context cultures place more importance on what is actually being said rather than the way in which it is said.^84^ Iver B. Neumann has therefore suggested that the expression of visual symbols will carry more weight in the communication in high-context cultures.^85^ For this reason, while there may be some overlap between states with a high-context national culture and ontological insecurity, it is difficult to pinpoint which of these factors that are decisive for if, why and how a state chooses to signal status and national pride.

Another signal sent through the staging of the participants’ backdrops was the placement of the EU flag in relation to the participating EU member states’ national flags. For instance, while the Italian flag and the EU flag were placed on either side of the Italian Prime Minister, Giuseppe Conte, the French flag almost completely obscured the EU flag placed behind President Macron. The German flag was also placed in front of the EU flag, but did not cover it to the same extent as the French flag did. While this flag placement may have simply been a coincidence, it could also be interpreted as a way to signal what ‘comes first’ – in other words, that national interests are prioritised over international cooperation with other EU member states. In this sense, the logic would be similar to why states would aim to break the ‘flag equality’ norm discussed above. Regardless of the intentions of the EU member states, showcasing the EU flag would also be a way of signalling that they are part of the second largest economy in the world,^86^ which would lend significant strength to their positions and the arguments made in the meeting. In this sense, the decision to display an EU flag is a way of signalling the states’ access to material capabilities, primarily in terms of economic power.

### Actors

**Figure 2.**
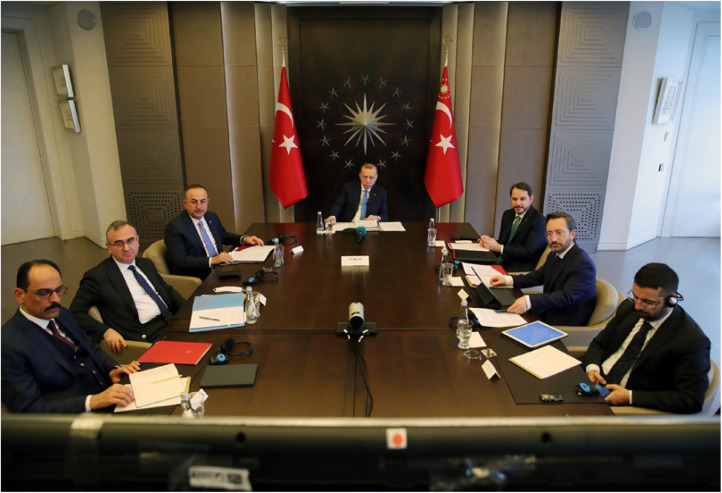
Photo of President Recep Tayyip Erdoğan and his advisors during the virtual G20 meeting. *Source*: @trpresidency

**Figure 3.**
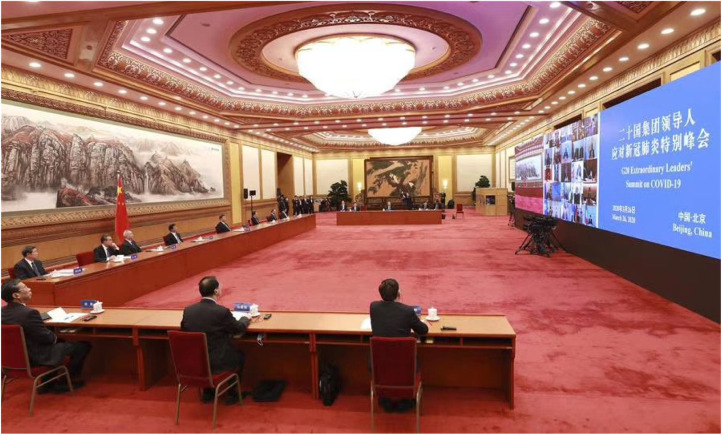
Photograph of Xi Jinping and his advisors during the virtual G20 meeting. *Source*: XNA.

Two main categories of actors participate at summit meetings: heads of state and government as well as their advisors. Together, these actors constitute each member state's delegation to a meeting. The nature of delegations, in terms of their level, composition, and size, is central to the recognition of actors in international diplomacy. Who is included in a delegation to an international summit is thus an indicator of both domestic and international social hierarchies and therefore essential to performances of mimicking an interaction order. Furthermore, cultural practices of inclusion or exclusion can reflect how diplomatic convention permits dialogue even in the absence of shared cultural assumptions.^87^ In lieu of a travelling delegation, the G20 member states signalled status through their staging of actors at the virtual meeting. The participating states showed large variations in this regard. Some states, such as Russia, France, Canada, Japan, and South Korea, chose to display their leader sitting alone, while other states included entire teams of advisors. Perhaps most strikingly, Turkey and Vietnam placed eight officials (all men) around their respective leaders, all centrally positioned (Figure 2).^88^ This can be contrasted with India, which placed five officials in the room, somewhat awkwardly off to the side so that they would not be seen behind Prime Minister Narendra Modi. Finally, China showcased a relatively small number of advisors sitting next to President Xi Jinping during the meeting. However, an image posted after the meeting by the state-run news agency XNA showed that there were at least 16 advisors present in the room (Figure 3).

The presence of a large number of advisors in the visual presentation of the state and its leader could be interpreted as a way to signal status, specifically by demonstrating reliability and material (economic) capabilities. In contrast to the signal that a leader sitting alone might send, a large number of advisors could be interpreted as a credible capacity to act competently – the more advisors in the room, the more likely it is that the leader will be perceived as taking well-grounded, competent decisions.^89^ A large number of participating advisors could also be seen as symbolic of material capabilities, as it signals that the state has a large administrative capacity. However, a large number of advisors could also adversely affect how others perceive a leader's trustworthiness.^90^ In this sense, the decision regarding how many actors that should be present in the visual presentation of the state and its leader could potentially be seen as a trade-off between acquiring status either through reliability and material capabilities, or through trust.

Another signal that could have an impact on a country's status is the role of the attending advisors and officials. For instance, the United States chose to place four officials next to Trump, including the Secretary of the Treasury, Steven Mnuchin, and the Chairman of the Joint Chiefs of Staff, Mark A. Milley (the highest-ranking General in the US Armed Forces). The choice of Milley was particularly interesting in this context, as no other state chose to have a military officer present at the meeting. In this sense, displaying Milley to the other participants is a fairly clear signal of the vast military superiority of the United States, which again draws on the status resource of material capabilities.

### Venue

The venues for international diplomacy are meticulously prepared. The choice of venue for a multilateral conference or summit is made with great care to provide an appropriate environment in which national predispositions are left behind and leaders are able to meet on common ground.^91^ In this particular context, the absence of a common summit venue required states to create their own venue through their choice of environment. The third way in which states signalled status was thus through the selection of the room in which the leaders sat during the meeting, as well as that room's decor. Like the other categories of symbolic action, there were large variations in terms of the characteristics of the rooms used by the leaders. While some leaders, such as Paul Kagame, the president of Rwanda; Pedro Sánchez, the prime minister of Spain; King Abdullah II of Jordan; and Ursula Von der Leyen, the president of the European Commission,^92^ chose to sit in relatively small, unembellished conference rooms, Xi Jinping was placed in front of a massive traditional painting in the East Hall of the Great Hall of the People in Beijing (Figure 3). The decision to use the East Hall was a clear signal of material capabilities and continuity – a large, grandiose room symbolises wealth and prestige, while the traditional artwork indicates that China has a long and rich history. Similarly, Macron chose to participate in the video conference from the Green Salon in the Elysée Palace, the official residence of the French president. The interior of the palace is remarkably ornate, with lavish decorations and furniture in the Baroque style, which is often associated with the *Ancien Régime*. For this reason, even one of the palace's more scaled down rooms would send a signal of the possession of both economic power and significant historical capital. The contrast between these choices also illustrates how some states view status competition and status seeking as more important than others. As Rwanda, Spain, and Jordan are not part of the G20, and the EU is not a sovereign state, the leaders of these states might not feel the same need to showcase that they have the same abovementioned status resources as the leaders of China and France.

The use of decorations, architectural monuments and ‘grandeur symbolism’ as a way to persuade other states and state leaders of the status of a state is arguably fundamental to the practice of modern-day diplomacy.^93^ The logic underpinning this type of persuasive symbolism is that prestige leads to the ability to project power. For this reason, the choice to showcase splendour, wealth, and cultural attractiveness can be seen as a conscious or subconscious way for these states to signal the ‘power a nation has or thinks it has, or wants other nations to believe it has’.^94^

Another significant choice of venue was that of the United States, which chose to participate from the White House Situation Room. The Situation Room has become a symbol of significant military capabilities, not least because of well-known photographs taken inside the room, such as the one taken of Barack Obama and his national security team during the raid on Osama bin Laden's compound in Abbottabad. For this reason, by choosing to participate from the Situation Room, Trump was advertently or inadvertently signalling that the United States possesses these military capabilities. In contrast, the choice – or rather, lack of choice – of locale by Justin Trudeau, who participated in the G20 meeting from his living room as a result of self-imposed quarantine following his wife's positive test for COVID-19 in early March 2020, did not exactly signal the military capabilities of Canada. The photographs from the meeting showing the Canadian prime minister sitting among his bookshelves instead had a rather familiar look, not least because of how commonplace video conferencing had become throughout the world by March and April 2020. This choice of venue could have been interpreted in multiple ways by the participating states and state leaders. One interpretation could be that Trudeau is signalling that he is taking the pandemic seriously, and therefore follows the same recommendations as all other citizens. Another could be that Trudeau is inadvertently signalling ontological security – that he/Canada is sufficiently content with their identity and position in the world that they have no need of trying to increase their relative status. In the context of multilateral summits, however, participating from your living room is also likely to signal a low degree of reliability. While a state leader could perhaps run their country from home, their perceived capacity to do so would be likely to decrease rather quickly.

### Props

The practice of diplomacy makes extensive use of symbols, rituals, and ceremonies that are often assisted by props.^95^ Props, originally theatrical properties, are the objects that connote diplomatic significance, such as clothes, official pins, decorations, and presented gifts that are used to enhance a performance. We identified three examples of the symbolic use of props during the virtual G20 meeting: (1) the choice of different coloured neckties among most of the participating leaders; (2) the decision to place hand sanitiser next to President Macron; and (3) the decision to place the Quran in front of King Salman of Saudi Arabia.

The effect of colour on human behaviour is a rather contradictory field of research. While some studies^96^ have found that wearing red clothing can lead to being perceived by others as aggressive and dominant, others have found that wearing red or blue has no effect on the perception of a politician's competence, dominance, and trustworthiness.^97^ However, since colours are associated with different situations, intentions, and beliefs, a blue tie is likely to be interpreted differently than a red one.^98^ Looking at the choice of ties at the virtual G20 meeting, all the participating male leaders wore either a red or a blue tie, with the exception of the president of Brazil, Jair Bolsonaro (striped tie), Modi and King Salman (no ties). The split between red and blue was fairly even: nine leaders chose a red tie, while 11 wore a blue tie. While the leaders’ choice of tie colour does not seem to have been systematic,^99^ the decision to wear either a blue or a red tie could potentially have signalled different status resources. For instance, a red tie would more likely be associated with aggression and dominance, and thus also a state's military capabilities, while a blue tie may signal the status resource of trustworthiness as a result of the colour blue being associated with openness.

**Figure 4.**
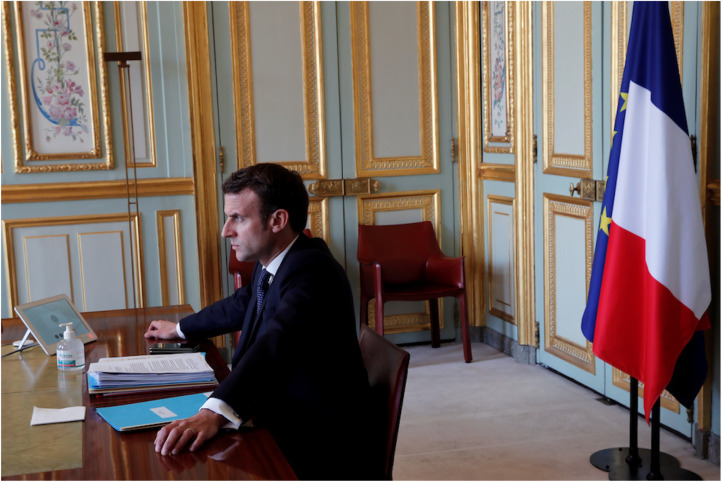
Photograph of President Macron (and his hand sanitiser) during the virtual G20 meeting. *Source*: Associated Press.

The second example of the use of props for status signalling during the meeting was the decision to place a bottle of hand sanitiser in front of President Macron (Figure 4). Regardless of whether this was an intentional placement, the result can be seen as a signal of the status resource of reliability. By explicitly demonstrating to the other leaders the importance of personal hygiene, Macron signalled a self-perception of competence and credibility in combating the spread of COVID-19. Given that Macron has portrayed France as in a ‘kinetic war’ against the virus, with him taking on the role of ‘commanding general’,^100^ it would not be surprising if Macron himself considered upholding the perception of his reliability as a crucial representation of his Self (including his status).

The final example of the use of props to signal status is the decision to place the Quran in front of King Salman, who also chaired the emergency meeting. Following the video conference, the Saudi Arabian Press Agency released three photographs of King Salman speaking and listening to the other G20 leaders. In the foreground of all three photographs was a green book with gilt edges, which we can only surmise to be the Quran. While the decision to place the Quran in front of King Salman may constitute the usual custom, it nonetheless symbolises a degree of continuity as it alludes to the long and rich history of the Muslim world. Given that religious symbols can carry a large symbolic value, not only for the values attached to the religion itself, but also as symbols for the identity of the state in question, the choice to place the Quran in front of King Salman can thus be seen as a signal of status based on the sanctity of traditions (historical capital). In addition, it could also be interpreted as a signal to other states that Saudi Arabia intends to follow the word of God rather than instrumental calculations in its foreign policy decision-making. In this sense, Saudi Arabia's relations with other states should be seen as predictable, which would increase its perceived status through the resource of trustworthiness – that is, consistent and credible action.

## Conclusions

Our analysis leads us to conclude that the absence of the protocol and routines that normally guide states’ participation during summit meetings generated new opportunities for states to manifest their status ambitions and self-perceptions through visual diplomacy. Drawing on Goffman's symbolism, we have suggested that the absence of protocol can be approached as a destabilised interaction order, which led the participants of the G20 virtual summit to adapt their performances through visual staging to make favourable impressions. While our analysis uncovered more variation than coherence in the participants’ efforts of staging their visual performances, our attention to status resources still uncovered significant patterns of status signalling. While these efforts could be argued to manifest conformity and lack of imagination, we have focused on how these visual performances where still breaking norms in the context of the otherwise visually controlled summit etiquette. The participants’ attempts to restore, or challenge the interaction order (by increasing one's status), reflected the perceptions of social value in terms of reliability, trustworthiness, material capabilities, and continuity. In this sense, the variation across the staged performances confirmed the role of visual diplomacy as a social practice in virtual summitry. The participants all engaged in status seeking behaviour by signalling in their visual performances. While we have maintained that these status resources and staging elements are not exhaustive, the fact that states and state leaders drew on them in order to signal status indicates that they (consciously or unconsciously) had a prior understanding of which visual cues matter in the social context of international summits.^101^

The virtual G20 meeting was unique in the sense that it was – as far as we know – the first high-level summit to take place via video conferencing that included all of the great powers. Unless the participating states coordinated their visual presentations prior to the meeting (the variation suggests they did not), they would have had no way of knowing how the other states would present themselves in this new setting. In other words, the states were forced into an ambiguous situation in which the main know-how to which they could resort was the diplomatic interaction order regarding status seeking – the resources, symbols, and signals that are perceived to have social value, and thus lead to status, in multilateral diplomacy.

In this particular context, visuality enhanced status signalling through the cultural significance of visual diplomatic performances. The visual cues that we identified were more explicit than what might have been expected from status seeking signals in speeches or texts, which require greater adaptation to protocol in the summit context. These findings are therefore relevant to a developed understanding of visual diplomacy, as our Goffmanesque framework has demonstrated that visual signalling not only matters for how states interact, but also offers specific ways in which to integrate visuality and visual methodology into sociological approaches to international relations. Moreover, the semiotic framework tends to draw symbolic meaning out of visual artefacts as a whole, thereby disavowing variation in signs. Our interactionist approach by way of status resources therefore contributes to provide a more nuanced understanding of the role of social interaction in status seeking through visual performances.

More specifically, the article explored how disruptions of diplomatic situations and settings, such as the one that arose following the COVID-19 outbreak in 2020, can have a significant impact on how states struggle to achieve and maintain status in world politics. When a new diplomatic setting is established, states and state leaders fall back on what they know: they act appropriately on the basis of the symbols and resources that they perceive to have social value in summit diplomacy, to increase their status by being seen in a favourable light. In this sense, understanding why states and state leaders seemingly have a near instinctive need to pursue status as well as seek to uphold a stable sense of Self, especially in times of crisis, deserves closer scrutiny in future research on status signalling and visual diplomacy.

## Footnotes

1 Christer Jönsson and Martin Hall, *Essence of Diplomacy* (New York, NY: Palgrave Macmillan, 2005); William C. Wohlforth, Benjamin de Carvalho, Halvard Leira, and Iver B. Neumann, ‘Moral authority and status in International Relations: Good states and the social dimension of status seeking’, Review of International Studies, 44:3 (2018), pp. 526–46.

2 Costas M. Constantinou, ‘Visual diplomacy: Reflections on diplomatic spectacle and cinematic thinking’, The Hague Journal of Diplomacy, 13:4 (2018), pp. 387–409.

3 William A. Callahan, ‘The politics of walls: Barriers, flows, and the sublime’, Review of International Studies, 44:3 (2018), pp. 456–81.

4 Raymond Cohen, *Theatre of Power: The Art of Diplomatic Signalling* (London, UK: Addison-Wesley Longman Ltd, 1987); Alisher Faizullaev, ‘Diplomacy and symbolism’, The Hague Journal of Diplomacy, 8 (2013), pp. 91–114.

5 Cohen, *Theatre of Power*.

6 Jönsson and Hall, *Essence of Diplomacy*.

7 Constantinou, ‘Visual diplomacy’, pp. 387–409.

8 Erving Goffman, *The Presentation of Self in Everyday Life* (Garden City, NY: Anchor, 1959); Erving Goffman, ‘The interaction order: American Sociological Association, 1982 presidential address’, American Sociological Review, 48:1 (1983), pp. 1–17; Iddo Tavory and Gary Alan Fine, ‘Disruption and the theory of the interaction order’, Theory and Society, 49:3 (2020), pp. 365–85.

9 In this article, we treat states, including their leaders, *as if* they are individual persons. This does not mean that we rule out the possibility that states actually *are* persons. For the purpose of this article, however, we do not believe that it is necessary to establish whether states actually are persons or that they simply act is if they are, as both perspectives should generate the same analysis and conclusions. Our view of states as (if) persons means that we view states as having identities, beliefs, and interests, as well as having the ability to consequently act upon those properties. This means that states have some degree of intentionality behind their actions and that they act as if they have feelings (such as ontological insecurity). Here, it is important to note that we do not believe that the intentionality of a state can or should be reduced to its individual members, including its leader. A state can act in ways that individuals cannot, and the identities, beliefs and interests of a state can thus also differ from those of any individual member. Nevertheless, it is reasonable to assume that individuals within a state will act in line with a degree of collective reason (a practical sense of what one should do as a member of that state), and that state leaders will thus be disposed, yet not determined, to act in line with the identities, beliefs and interests of the state. For a longer discussion on the role of intentionality in defining the state as a person and for an argument that state persons are ‘real’, see Alexander Wendt, ‘The state as person in international theory’, Review of international Studies, 30:2 (2004), pp. 289–316. Ku and Mitzen treat states in a similar way in their study of system trust in summitry, see Minseon Ku and Jennifer Mitzen, ‘Trusting Anarchy: Summits and the Production of State Personhood’, paper presented at the political psychology seminar, Lund University, 6 May 2021.

10 Tristen Naylor, ‘All that's lost: The hollowing of summit diplomacy in a socially distanced world’, The Hague Journal of Diplomacy, 15 (2020), pp. 583–98.

11 Kristin Anabel Eggeling and Rebecca Adler-Nissen, ‘The synthetic situation in diplomacy: Scopic media and the digital mediation of estrangement’, *Global Studies Quarterly*, 1 (2021).

12 Goffman, *The Presentation of Self in Everyday Life*; Goffman, ‘The interaction order’, pp. 1–17.

13 Patrick Thaddeus Jackson and Daniel H. Nexon, ‘Relations before states: Substance, process and the study of world politics’, European Journal of International Relations, 5:3 (1999), pp. 291–332; Rebecca Adler-Nissen, ‘Stigma management in International Relations: Transgressive identities, norms, and order in international society’, International Organization, 68 (2014), pp. 143–76; Marina G. Duque, ‘Recognizing international status: A relational approach’, International Studies Quarterly, 62 (2018), pp. 577–92; T. V. Paul, Deborah Welch Larson, and William C. Wohlforth (eds), *Status in World Politics* (Cambridge, UK: Cambridge University Press, 2014); Ayse Zarakol, *After Defeat: How the East Learned to Live with the West* (Cambridge, UK: Cambridge University Press, 2010); Ayse Zarakol, ‘What made the modern world hang together: Socialisation or stigmatisation’, *International Theory*, 6 (2014), pp. 311–32.

14 Goffman, ‘The interaction order’, pp. 1–17; Frank Schimmelfennig, ‘Goffman meets IR: Dramaturgical action in international community’, *International Review of Sociology*, 12:3 (2002), pp. 417–37; Rebecca Adler-Nissen, ‘Diplomacy as Impression Management: Strategic Face-Work and Post-Colonial Embarrassment’, prepared for the CIPSS Speaker Series on International Security and Economy, McGill University (2012).

15 Graham Spencer, *The Media and Peace* (London, UK: Palgrave Macmillan, 2005); Constantinou, ‘Visual diplomacy’, pp. 387–409; Iver B. Neumann, ‘The evolution of visual diplomacy’, in *Diplomatic Tenses* (Manchester, UK: Manchester University Press, 2020), pp. 46–71.

16 Rebecca Adler-Nissen and Alexei Tsinovoi, ‘International misrecognition: The politics of humour and national identity in Israel's public diplomacy’, European Journal of International Relations, 25:1 (2019), pp. 3–29.

17 Todd Hall and Keren Yahri-Milo, ‘The personal touch: Leaders’ impressions, costly signaling, and assessments of sincerity in international affairs’, *International Studies Quarterly*, 56:3 (2012), pp. 560–73; Kai Quek, ‘Are costly signals more credible? Evidence of sender-receiver gaps’, *The Journal of Politics*, 78:3 (2016), pp. 925–40; Seanon S. Wong, ‘Emotions and the communication of intentions in face-to-face diplomacy’, European Journal of International Relations, 22:1 (2016), pp. 144–67; Seanon S. Wong, ‘Mapping the repertoire of emotions and their communicative functions in face-to-face diplomacy’, *International Studies Review*, 22 (2020), pp. 77–97; Constance Duncombe, ‘Twitter and transformative diplomacy: Social media and Iran–US relations’, *International Affairs*, 93 (2017), pp. 545–62.

18 Neumann, ‘The evolution of visual diplomacy’, pp. 46–71.

19 Marc Rancière, *The Politics of Aesthetics* (London, UK and New York, NY: Bloomsbury Publishing, 2013).

20 Roland Bleiker (ed.), *Visual Global Politics* (London, UK and New York, NY: Routledge, 2018).

21 Faizullaev, ‘Diplomacy and symbolism’, pp. 91–114.

22 Michael J. Shapiro, *Cinematic Geopolitics* (London, UK: Routledge, 2008), p. 92.

23 Neumann, ‘The evolution of visual diplomacy’, pp. 46–7.

24 Constantinou, ‘Visual diplomacy’, pp. 387–409.

25 Roland Bleiker, ‘Pluralist methods for visual global politics’, *Millennium*, 43 (2015), pp. 872–90.

26 Jan Melissen, *Summit Diplomacy Coming of Age* (Netherlands Institute of International Relations, The Hague, 2003).

27 An early example of the use of visual cues to signal status was seen at the Potsdam Conference in 1945, where additional doors were added to the summit meeting room in order for the Big Three to be able to enter the room simultaneously, thereby signalling equal status.

28 Melissen, *Summit Diplomacy Coming of Age*.

29 Ilan Manor, *The Digitalization of Public Diplomacy* (Basingstoke, UK: Palgrave Macmillan 2019).

30 Cohen, *Theatre of Power*.

31 Robert D. Putnam, ‘Diplomacy and domestic politics: The logic of two-level games’, *International Organization*, 42 (1988), pp. 427–60.

32 Jönsson and Hall, *Essence of Diplomacy*.

33 Hall and Yahri-Milo, ‘The personal touch’, pp. 560–73; Quek, ‘Are costly signals more credible?’, pp. 925–40; Wong, ‘Emotions and the communication of intentions in face-to-face diplomacy’, pp. 144–67; Wong, ‘Mapping the repertoire of emotions and their communicative functions in face-to-face diplomacy’, pp. 77–97; Duncombe, ‘Twitter and transformative diplomacy’, pp. 545–62.

34 Costas M. Constantinou, ‘Diplomacy’, in Bleiker (ed.), *Visual Global Politics*, pp. 104–10; Andrew F. Cooper and. Jérémie Cornut, ‘The changing practices of frontline diplomacy: New directions for inquiry’, *Review of International Studies*, 45 (2019), pp. 300–19; Elsa Hedling and Niklas Bremberg, ‘Practice approaches to the digital transformations of diplomacy: Toward a new research agenda’, *International Studies Review* (2021).

35 Elsa Hedling, ‘Storytelling in EU public diplomacy: Reputation management and recognition of success’, *Place Branding and Public Diplomacy* (2019), pp. 1–10.

36 Noé Cornago, *Plural Diplomacies: Normative Predicaments and Functional Imperatives* (Leiden, NL: Martinus Nijhoff Publishers, 2013).

37 Ilad Manor and Elad Segev, ‘America's selfie: How the US portrays itself on its social media accounts’, in Corneliu Bjola and Marcus Holmes (eds), *Digital Diplomacy* (London, UK: Routledge, 2015), pp. 103–22; Constance Duncombe, ‘Digital diplomacy: Emotion and identity in the public realm’, *The Hague Journal of Diplomacy*, 14 (2019), pp. 102–16; Karin Aggestam, Annika Bergman Rosamond, and Elsa Hedling, ‘Feminist digital diplomacy and foreign policy change in Sweden’, *Place Branding and Public Diplomacy* (2021), pp. 1–11.

38 Constantinou, ‘Diplomacy’, pp. 104–10.

39 Iver B. Neumann, ‘Diplomacy and the arts’, in Costas M. Constantinou, Pauline Kerr, and Paul Sharp (eds), *The SAGE Handbook of Diplomacy* (Thousand Oaks, CA: SAGE Publishing 2016), pp. 114–22.

40 Alexander Wendt, ‘Why a world state is inevitable’, *European Journal of International Relations*, 9 (2003), pp. 491–542.

41 Deborah Welch Larson and Alexei Shevchenko, ‘Managing rising powers: The role of status concerns’, in Paul, Larson, and Wohlforth (eds), *Status in World Politics*, pp. 33–57.

42 Ibid.

43 Anne L. Clunan, ‘Why status matters in world politics’, in Paul, Larson, and Wohlforth (eds), *Status in World Politics*, pp. 273–96.

44 Max Weber, *Economy and Society: An Outline of Interpretive Sociology* (New York, NY: Bedminster Press, 1978); David A. Lake, *Hierarchy in International Relations* (New York, NY: Cornell University Press, 2011).

45 Jennifer L. Miller, Jacob Cramer, Thomas J. Volgy, Paul Bezerra, Megan Hauser, and Christina Sciabarra, ‘Norms, behavioral compliance, and status attribution in international politics’, *International Interactions*, 41 (2015), pp. 779–804.

46 Duque, ‘Recognizing international status’, pp. 577–92.

47 Zarakol, *After Defeat*; Zarakol, ‘What made the modern world hang together’, pp. 311–32; Ayse Zarakol, ‘States and ontological security: A historical rethinking’, *Cooperation and Conflict*, 52 (2017), pp. 48–68.

48 Vincent Pouliot, ‘Setting status in stone: The negotiation of international institutional privileges’, in Paul, Larson, and Wohlforth (eds), *Status in World Politics*, pp. 192–215.

49 Larson and Shevchenko, ‘Managing rising powers’, pp. 33–57.

50 We recognise the limits of Goffman's approach to performativity as instrumental to social order rather than constitutive of social change. Since we study visual performances as reflections of routines of social interaction rather than the effect of change, Goffman's dramaturgy still suffices to provide an interactionist perspective on visual semiotics.

51 Goffman, *The Presentation of Self in Everyday Life*.

52 Michelle Murray, *The Struggle for Recognition in International Relations: Status, Revisionism, and Rising Powers* (Oxford, UK: Oxford University Press, 2018).

53 Goffman, *The Presentation of Self in Everyday Life*.

54 Ibid.

55 Vincent Pouliot, *International Pecking Orders: The Politics and Practice of Multilateral Diplomacy* (Cambridge, UK: Cambridge University Press, 2016), p. 61.

56 Goffman, *The Presentation of Self in Everyday Life*.

57 Cohen, *Theatre of Power*.

58 Goffman, *The Presentation of Self in Everyday Life*.

59 David E. Banks, ‘The diplomatic presentation of the state in international crises: Diplomatic collaboration during the US-Iran hostage crisis’, *International Studies Quarterly*, 63 (2019), pp. 1163–74.

60 John Freedman, ‘Status insecurity and temporality in world politics’, *European Journal of International Relations*, 22:4 (2016), pp. 797–822; Clunan, ‘Why status matters in world politics’, pp. 273–96; Miller et al., ‘Norms, behavioral compliance, and status attribution in international politics’, pp. 779–804.

61 Erving Goffman, ‘Symbols of class status’, *The British Journal of Sociology*, 2 (1951), pp. 294–304.

62 Alastair Iain Johnston, *Social States: China in International Institutions, 1980–2000* (Princeton, NJ: Princeton University Press, 2008).

63 Pouliot, ‘Setting status in stone’, pp. 192–215.

64 Deborah Welch Larson and Alexei Shevchenko, *Quest for Status: Chinese and Russian Foreign Policy* (New Haven, CT: Yale University Press, 2019).

65 Danielle L. Lupton, ‘Reexamining reputation for resolve: Leaders, states, and the onset of international crises’, *Journal of Global Security Studies*, 3 (2018), pp. 198–216.

66 Hall and Yahri-Milo, ‘The personal touch’, pp. 560–73.

67 Pouliot, ‘Setting status in stone’, pp. 192–215.

68 Robert Gilpin, *War and Change in World Politics* (Cambridge, UK: Cambridge University Press, 1981); Deborah Welch Larson and Alexei Shevchenko, ‘Status seekers: Chinese and Russian responses to US primacy’, *International Security*, 34 (2010), pp. 63–95; William C. Wohlforth, ‘Unipolarity, status competition, and great power war’, *World Politics*, 61 (2009), p. 28.

69 Weber, *Economy and Society.*

70 Edward Keene, ‘Social status, social closure and the idea of Europe as a “normative power”’, *European Journal of International Relations*, 19 (2013), pp. 939–56.

71 Zarakol, *After Defeat*.

72 This also entails that we acknowledge the possibility of alternative interpretations than the ones we offer. However, we aim to remedy this by making as sound arguments as possible for why the signals that we identify should be perceived by the summit participants as having social and signalling value.

73 Christer Jönsson, ‘Diplomacy, communication and signalling’, in Constantinou, Kerr, and Sharp (eds), *The SAGE Handbook of Diplomacy*, pp. 79–91.

74 Mieke Bal and Norman Bryson, ‘Semiotics and art history’, *The Art Bulletin*, 73 (1991), pp. 174–208.

75 Gillian Rose, *Visual Methodologies: An Introduction to Researching with Visual Materials* (London, UK: SAGE Publishing, 2016).

76 Ibid.

77 Goffman, *The Presentation of Self in Everyday Life*; Cohen, *Theatre of Power*.

78 Faizullaev, ‘Diplomacy and symbolism’, pp. 91–114.

79 Daryl Copeland, *Guerrilla Diplomacy: Rethinking International Relations* (Boulder, CO: Lynne Rienner Publishers 2009).

80 Faizullaev, ‘Diplomacy and symbolism’, pp. 91–114.

81 Zarakol, *After Defeat*.

82 Raymond Cohen, *Negotiating across Cultures: International Communication in an Interdependent World* (Washington, DC: US institute of Peace Press, 1997).

83 Edward T. Hall, *Beyond Culture* (New York, NY: Anchor Press/Doubleday, 1976).

84 Faizullaev, ‘Diplomacy and symbolism’, pp. 91–114.

85 Neumann, ‘The evolution of visual diplomacy’, pp. 46–71.

86 International Monetary Fund, ‘World Economic Outlook Database' (Washington, DC: 2019).

87 Cohen, *Theatre of Power*.

88 The current holder of the ASEAN chair (Vietnam in 2020) is a permanent guest invitee at G20 meetings.

89 For an overview of the literature on the effect of group size on performance, see Glenn E. Littlepage, ‘Effects of group size and task characteristics on group performance: A test of Steiner's model’, *Personality and Social Psychology Bulletin*, 17 (1991), pp. 449–56.

90 Studies in social psychology have found that group size is negatively correlated with trust, see Marilynn B. Brewer, ‘The social self: On being the same and different at the same time’, *Personality and Social Psychology Bulletin*, 17 (1991), pp. 475–82; Bas Denters, ‘Size and political trust: Evidence from Denmark, the Netherlands, Norway, and the United Kingdom’, *Environment and Planning C: Government and Policy*, 20:6 (2002), pp. 793–812.

91 Klaus L. Aurisch, ‘The art of preparing a multilateral conference’, Negotiation Journal, 5 (1989), pp. 279–88; Ronald A. Walker, *Multilateral Conferences: Purposeful International Negotiation* (London, UK: Palgrave Macmillan 2004); G. R. Berridge, *Diplomacy: Theory and Practice* (Basingstoke, UK: Palgrave MacMillan, 2015).

92 Rwanda, Spain, and Jordan were all guest invitees at the meeting. Despite the fact that it is not a sovereign state, the European Union is one of the twenty ordinary members of the G20. Peter Debaere, ‘The European Union and the Gx system’, in Peter Debaere (ed.), *EU Coordination in International Institutions: Policy and Process in Gx Forums* (London, UK: Palgrave Macmillan, 2015), pp. 10–19.

93 Faizullaev, ‘Diplomacy and symbolism’, pp. 91–114.

94 Hans Morgenthau, *Politics Among Nations: The Struggle for Power and Peace* (New York, NY: Knopf, 1948).

95 Deepak Nair, ‘Saving face in diplomacy: A political sociology of face-to-face interactions in the Association of Southeast Asian Nations’, *European Journal of International Relations*, 25:3 (2019), pp. 672–97; Banks, ‘The diplomatic presentation of the state in international crises’, pp. 1163–74.

96 Diana Wiedemann, D. Michael Burt, Russell Hill, and Robert Barton, ‘Red clothing increases perceived dominance, aggression and anger’, *Biology Letters*, 11 (2015); Russell Hill and Robert Barton, ‘Red enhances human performance in contests’, *Nature*, 435 (2005), p. 293.

97 Robin S. S. Kramer, ‘The red power (less) tie: Perceptions of political leaders wearing red’, *Evolutionary Psychology*, 14 (2016).

98 Red is commonly associated with danger and mistakes, while blue is associated with openness, peace, and tranquillity; see Mehta and Zhu, ‘Blue or red? Exploring the effect of color on cognitive task performances’, *Science*, 323 (2009), pp. 1226–9.

99 For instance, no clear correlation could be found between the size of a state and the choice of tie colour.

100 Rym Momtaz, ‘Inside Macron's coronavirus war’, *Politico* (12 April 2020).

101 The notion that state leaders are aware of, and react to, the signals being sent through visual cues was emphasised in a recent interview with the former prime minister of Sweden, Carl Bildt: ‘I've been irritated at the fact that [the Swedish government] has removed the EU flag during their press conferences. When they have a video conference with other EU leaders, they bring the EU flag in and then they take it out again. Those types of things are noticed.’ Carl Hultin, ‘Bildt: Tegnell ska inte sätta sig på höga hästar’, *Svenska Dagbladet* (10 May 2020).

